# Research Progress of Heavy Ion Radiotherapy for Non-Small-Cell Lung Cancer

**DOI:** 10.3390/ijms23042316

**Published:** 2022-02-19

**Authors:** Siqi Liang, Guangming Zhou, Wentao Hu

**Affiliations:** State Key Laboratory of Radiation Medicine and Protection, School of Radiation Medicine and Protection, Collaborative Innovation Center of Radiological Medicine of Jiangsu Higher Education Institutions, Soochow University, Suzhou 215123, China; 1913401015@stu.suda.edu.cn

**Keywords:** heavy ion radiotherapy, carbon ion radiotherapy, non-small-cell lung cancer, curative effect

## Abstract

Non-small-cell lung cancer (NSCLC) has a high incidence and poses a serious threat to human health. However, the treatment outcomes of concurrent chemoradiotherapy for non-small-cell lung cancer are still unsatisfactory, especially for high grade lesions. As a new cancer treatment, heavy ion radiotherapy has shown promising efficacy and safety in the treatment of non-small-cell lung cancer. This article discusses the clinical progress of heavy ion radiotherapy in the treatment of non-small-cell lung cancer mainly from the different cancer stages, the different doses of heavy ion beams, and the patient’s individual factors, and explores the deficiency of heavy ion radiotherapy in the treatment of non-small-cell lung cancer and the directions of future research, in order to provide reference for the wider and better application of heavy ion radiotherapy in the future.

## 1. Non-Small-Cell Lung Cancer

Lung cancer is a kind of malignant tumor originating from the bronchial mucosa or glands. According to the statistics of the World Health Organization, there were more than 2.2 million new cases of lung cancer in 2020, accounting for 11.4% of all newly diagnosed cancers, ranking second, and the mortality rate was 18%, ranking first, and it is still increasing year by year [1]. Among them, there were more male patients than female patients, and the high incidence group was over 75 years old [2]. In China, the incidence of lung cancer is 35.13/100,000, and the mortality is 28.57/100,000, ranking first among all cancers. The incidence and mortality of lung cancer remain low in the age group under 40 years old, but the incidence and mortality of lung cancer rise sharply and reach a peak in the age group from 80 to 84 years old [3]. Air pollution, smoking, other bad living habits, as well as genetic factors, are the main causes of lung cancer. According to pathological classification, lung cancer can be divided into small-cell lung cancer (SCLC) and non-small-cell lung cancer (NSCLC), of which NSCLC accounts for approximately 80–90% of lung cancer. Therefore, the treatment of NSCLC is of great significance.

## 2. Heavy Ion Radiotherapy

Although the current treatment of lung cancer is comprehensive and based on surgery, due to the lack of awareness of regular physical examination, many patients at the time of diagnosis are already in the late stage, which means that they are not suitable for surgery [4]. As a main treatment means used in lung cancer, radiotherapy has a long history. In conventional X-ray radiotherapy, it is difficult to transfer enough dose to the tumor part since the tumor tissue is surrounded by normal lung tissue and the electron density is low [5], which means that X-ray treatment requires a higher dose to achieve a certain therapeutic effect, which may increase the probability of radiation damage [6]. As a new radiotherapy method, heavy ion radiotherapy has better efficacy and fewer adverse reactions compared with photon radiotherapy. In stage I inoperable NSCLC, 5-year overall survival for conventional radiotherapy (CRT) (20%) was significantly lower than that for carbon ion radiotherapy (CIRT) (42%) [7]. Kubo et al. found that the residual dose of CIRT was lower than that of X-ray radiotherapy in normal lung tissue, spinal cord, bone, and esophagus at the same radiation dose. The main adverse event, radiation pneumonia, occurred in 5.2% of patients receiving CIRT and 8.5% of patients receiving X-rays. Under the same irradiation dose conditions, the target dose of heavy ion (mainly carbon ion) treatment is higher, and the damage to surrounding normal tissues is less [8]. Wink et al. compared the effects of carbon ions, protons, and photons on stage I NSCLC. A total of 24 patients were treated with a dose prescription of 60 Gy, delivered in eight fractions (fr). The results showed that the mean dose of protons and carbon ions was lower than that of photons, and the dose of carbon ions to the lung, heart, and mediastinum was the lowest; that is, the dangerous organ dose of carbon ions was lower [9]. Ebara et al. compared dosimetry between stereotactic body radiotherapy (SBRT) with CIRT in 13 stage I NSCLC patients. In the CIRT group, prescription of the dose and fractionation for stage IA and IB were 52.8 Gy/4 fr and 60.0 Gy/4 fr, respectively. SBRT group was given the same dose as CIRT group. The results showed that the normal lung tissue dose in the CIRT group was significantly lower than that in the SBRT group. Even for larger tumors, the normal lung tissue dose of CIRT was lower and its target conformity was better [10]. Iwata et al. studied 70 T2a/bN0M0 NSCLC patients who received proton or CIRT. Forty-three of them received proton radiotherapy, and 27 received CIRT. The 4-year overall survival rate and local control rate were 58% and 75%, respectively. Grade 3 pulmonary adverse reactions were only observed in two patients, which was better than SBRT [11].

Taking CIRT as an example, this paper will review the biological effects of heavy ions, the clinical progress of heavy ions in the treatment of NSCLC, and future research directions to provide a reference for the application of heavy ions in the treatment of NSCLC.

## 3. Heavy Ions and Their Biological Effects

Heavy ions refer to ions with atomic numbers greater than two (helium); commonly used are ^12^C and ^56^Fe. Heavy ions have quite unique biological effects. First, charged heavy ions can produce a fairly high linear energy transfer (LET) when interacting with human tissues and organs, which means that heavy ions can transfer higher energy to tissues when traveling the same distance [12,13]. Second, the ratio of direct action is higher when the interaction between heavy ions and human tissue occurs, and a lower oxygen enhancement ratio (OER) and higher relative biological effectiveness (RBE) will appear, which is on the order of threefold greater than photon radiotherapy [14]. When heavy ions interact with DNA, they cause unique clustered DNA damage that is difficult to recover through the cell’s own DNA repair pathway, leading to a high mortality rate of tumor cells, which is also effective in some radiation-resistant tumors. Like protons and photons, heavy ions can induce changes in the tumor cell membrane surface, such as increased ecto-calreticulin exposure, which makes tumor cells more easily recognized and cleared by the immune system and plays an important role in the activation of antitumor immunity [15]. Finally, compared with low LET radiations, the deposition of the energy of heavy ions in the material has a great advantage. The energy stored by heavy ions in tissues varies with depth as follows: In the range of the flat region, energy deposition remains at a low dose with increasing depth; while at the end of the range, energy increases suddenly and then decreases rapidly. This increased energy is referred to as the Bragg peak. The significance of the Bragg peak is that the physician can control the energy of the incident particle to determine the depth of the Bragg peak, so that the maximum deposition of energy occurs at the site of the lesion, and less deposition occurs at the site of the normal tissues. However, the Bragg peak has a narrow half-height width of just a few millimeters, which means that the maximum deposition energy lasts only a short time. Therefore, spread-out Bragg peak (SOBP) is often used clinically [16,17]. Although the energy at the incident site will increase, it is still lower than the maximum deposited energy, and the treatment effect is guaranteed while the damage to normal tissues is small [8].

## 4. Radiobiological Effects of Heavy Ions on Lung Cancer Cells and the Underlying Molecular Mechanisms

Currently, there has been much progress in the mechanistic studies of the radiobiological effects of heavy ions on NSCLC cells, which are mainly concentrated on the following aspects:

1. Killing effects of heavy ions on NSCLC cells. Heavy ions cause apoptosis, necrosis, and senescence of cancer cells by causing complex DNA double-strand breaks (DSBs), which is also the main beneficial effect of heavy ions in cancer treatment. Low-LET radiation (photon) causes simple DNA damage within one or two circles of the DNA helix. In contrast, high-LET charged particle radiation mainly causes clustered DNA damage, which is characterized by multiple adjacent DNA lesions [18,19]. Using high resolution transmission electron microscopy (TEM) and gold-labeled DNA repair factors, it was found that the clustering of DSBs in heterochromatin following high-LET irradiation perturbed efficient DNA repair, leading to greater biological effectiveness versus that of low-LET irradiation [20]. This conclusion has also been confirmed in clinical CIRT. Using advanced high-resolution microscopy with deconvolution, Oike et al. observed the formation of complex DSBs in a human tumor clinically treated with CIRT, rather than X-ray radiotherapy [21]. DNA damage caused by heavy ions is more complex than that caused by X-rays, making it difficult for the DNA repair pathway to function effectively [22]. In other words, inhibition of DNA repair can enhance the effect of heavy ion therapy. Nakajima et al. investigated the involvement of the DNA damage signaling factors ataxia telangiectasia mutated (ATM), RING finger protein 8 (RNF8), and RNF168 in cells after high LET carbon ion irradiation; the results suggest that inhibition of RNF8 activity or its downstream pathway may enhance the efficacy of CIRT [23]. Yang et al. found that inhibition of DNA-PKcs enhanced radiosensitivity and increased the expression levels of ATM and ATR in NSCLC cells exposed to carbon ion irradiation, implying a role for DNA-PKcs in DNA damage repair signaling induced by carbon ions [24]. For photon radiation, they can indirectly damage DNA or other cellular structures by producing reactive oxygen species (ROS). This effect requires the participation of oxygen in the tumor microenvironment, so low-LET photons have poor killing effect on cancer cells under hypoxia conditions [25]. However, DNA damage induced by carbon ions does not depend on ROS production [26]. One explanation is the increased expression of hypoxia inducible factor 1α (HIF-1α) after photon irradiation [27], while heavy ions do not, so they work well in hypoxia conditions. Klein et al. irradiated A549 and H1437 cells with different doses of photons or carbon ions under hypoxic (1% O_2_) or normoxic (21% O_2_) conditions respectively. The inhibitors of DNA-PK and ATM were used in parallel. The results showed that DNA-PK inhibition combined with carbon irradiation was most effective in killing NSCLC cells under hypoxic conditions [28]. Carbon ions can cause clustered DNA damage, which is difficult to repair. This characteristic, which is superior to photons, endows carbon ions with stronger killing effect on NSCLC cells, even those that are hypoxic and radio-resistant. Further, many non-coding RNAs (ncRNAs) such as microRNAs (miRNAs), long non-coding RNAs (lncRNAs), and circular RNAs (circRNAs) play a regulatory role in the killing of lung cancer cells by heavy ions. Liu et al. found that circRNA ZNF208 significantly enhanced the resistance of NSCLC cells to X-rays, but the sensitivity to carbon ions did not change [29]. In addition to circRNA ZNF208, the down-regulation of lncRNA H19 also increases the sensitivity of NSCLC cells to heavy ions [30]. Therefore, these ncRNAs might function as a potential therapeutic target to enhance the efficacy of heavy ion radiotherapy for NSCLC. Despite high RBE and high tumor-killing ability of carbon ions, the tumor recurrence after CIRT is often observed, suggesting the presence of a subset of tumor cells resistant to CIRT. Darwis et al. identified a pivotal role for FGFR signaling in cancer cell survival through CIRT, and found that inhibition of FGFR using pan-FGFR inhibitor LY2874455 sensitized multiple NSCLC cell lines to carbon ions, which may be useful in the sensitization of CIRT-resistant cancers [31]. Besides, Amornwichet et al. found *EGFR*-mutant NSCLC cells, rather than *KRAS*-mutant NSCLC cells, showed low RBE of carbon ions over X-rays, indicating the potential of *EGFR* mutation status as a predictor of cellular response to CIRT [32]. These results suggest that clarification of the governing factors and signaling pathways in cellular response to carbon ion irradiation will help to further improve the treatment efficacy of CIRT.
2. Effects of heavy ions on invasion and metastasis of lung cancer cells. Photon irradiation, rather than heavy ion irradiation, was found to significantly induce expression of matrix metalloproteinases (MMPs), stem cell factor (SCF), and β1-integrin, which promote angiogenesis and cancer cell metastasis [33,34,35]. Liu et al. irradiated A549 cells with carbon ions and X-rays. Carbon-ion irradiation at 1 Gy significantly reduced vascular endothelial growth factor (VEGF) and basic fibroblast growth factor (bFGF) levels and inhibited endothelial cell invasion and tube formation, suggesting inhibiting potential of angiogenesis by carbon ions [36]. Akino et al. irradiated A549 and EBC-1 lung cancer cells with 290 MeV/u carbon ion beams and 4 MeV X-rays. The results showed that carbon ion irradiation inhibited the migration and invasion of A549 and EBC-1 cells more effectively than X-rays. In addition, they found that carbon ion irradiation alone induced downregulation of ANLN expression in NSCLC cells, which is downstream of PI3K/Akt signaling and positively associated with tumor metastasis [37]. On this basis, Ogata et al. found that low dose carbon ion irradiation also reduced the level of phosphorylated Akt compared with untreated control group, while photon irradiation did not, suggesting that carbon ion irradiation can effectively inhibit the metastatic potential of A549 cells by suppressing the PI3K/Akt signaling pathway [38]. In addition, Kamlah et al. found that irradiation of A549 cells with X-rays (6 Gy) but not carbon ions (2 Gy) resulted in a significant increase in blood vessel density through increased expression of SCF and subsequently phosphorylation of c-Kit [35]. In summary, carbon ions have a distinct advantage over X-rays in preventing angiogenesis and the spread of tumors.
3. Effects of heavy ions on immunogenicity of lung cancer cells. Radiation not only has an immunostimulatory effect, but also shows an immunosuppressive effect. Therefore, it is important to understand the immunomodulatory properties of radiation to enhance the curative effect of radiotherapy [39,40,41]. The change of immunogenicity induced by heavy ions is key in the regulation of tumor immunity. Heavy ions cause tumor cells to die in different ways and release pro-inflammatory cytokines; chemokines; tumor antigens; and other danger signals, called damage-associated molecular patterns (DAMPs). DAMPs can activate the immune system, and immune cells are attracted to the area where the tumor is located [42]. Some progress has been made in the study of heavy ion-induced DAMPs. Ran et al. used ELISA to detect the levels of HMGB1, IL-10, and TGF-β in A549, H520, and Lewis Lung Carcinoma (LLC) cell lines under different “time windows” and “dose windows” after X-ray or carbon ion irradiation. The results showed that both X-rays and carbon ions promoted HMGB1, IL-10, and TGF-β levels in a time-dependent manner, and only X-rays increased the HMGB1 level in a dose-dependent manner. In addition, carbon ions increased higher HMGB1 levels compared with X-rays, while the levels of immunosuppressive factors IL-10 and TGF-β were relatively reduced. These results suggest that carbon ions may be superior to conventional X-rays in inducing immune-enhancing effects [43]. Huang et al. found that carbon ions promoted the cell surface translocation of calreticulin more strongly than protons and photons at 2 and 4 Gy, which plays a pivotal role in activating anti-tumor immunity [15]. In addition, Wang et al. found that carbon ions noticeably induced *Klrk1* gene expression and activated the NKG2D/NKG2D-Ls pathway in a murine Lewis lung cancer model, which were tightly related to the functional status of NK cells. CIRT combined with Treg inhibition significantly increased the infiltration and function of NK cells and prolonged the survival of cancer-bearing mice [44]. Compared with photons, heavy ions can play a better role in induction of tumor immunity, rendering the combination of heavy ion radiotherapy and immunotherapy a promising therapy.

In conclusion, compared with X-rays, heavy ions can cause DNA damage that is more difficult to repair, have a stronger killing effect on lung cancer cells, and play a unique role in tumor immunity. Besides, carbon ions have a distinct advantage over X-rays in preventing angiogenesis and the spread of tumors. Although a lot of work aiming at elucidating the molecular mechanisms underlying the radiobiological effects of heavy ions has been conducted, much remains to be done. First, repair of clustered DNA lesions induced by carbon ion radiation is not well understood. Failure to repair clustered DNA damage leads to more abnormal mitosis and mitotic catastrophe. However, it is not clear whether homologous recombination (HR) or non-homologous end joining (NHEJ) plays a more important role in the repair of clustered DNA damage [45,46]. Second, it is important to understand the epigenetic regulation of the killing effects of heavy ions in lung cancer cells. It has been shown that individual radiosensitivity is tightly related to the epigenetic regulation of genes participating in chromatin remodeling and DNA damage repair [47]. Therefore, the study of the regulatory mechanisms of these genes will provide important theoretical basis for more precise and effective heavy ion radiotherapy. Last but not least, the molecular mechanism underlying immunogenicity changes induced by heavy ions is still unclear. Also, there is no clear conclusion about the effect of dose rate and dose fractionation on immunogenicity induced by heavy ions. In a word, there is no doubt that the further development of these studies will greatly improve the efficacy of heavy ion radiotherapy and reduce its side effects.

## 5. Research Progress on Heavy Ion Therapy for Non-Small-Cell Lung Cancer

CIRT showed high efficacy in different stages of NSCLC. For stage I NSCLC, Miyamoto et al. treated 79 patients with CIRT, and the results showed that the 5-year local control rate of patients reached 90%, the 5-year survival rate was 68%, and the overall survival rate was 45%, while the 5-year survival rate of patients receiving traditional radiotherapy was only 15–30% [48]. In another study, Miyamoto et al. studied 146 patients with stage I NSCLC. The local control rate was 82%, while the overall survival rate was 59%. Only a small number of patients developed radioactive pneumonia (2.1%) [49]. Baba et al. studied the treatment outcomes of 129 patients with an average age of 74.5 years. The dose project was a fixed total dose of 72 GyE in nine fractions over 3 weeks, 52.8 GyE for stage IA, 60 GyE for stage IB. The results showed that the local control rate of all lesions was 94.7%, the 5-year survival rate of stage IA was 68.7%, and the 5-year survival rate of stage IB was 46.4%. No skin toxicity was observed, and no adverse reactions beyond grade 3 were observed [50]. Shioyama et al. conducted a retrospective analysis on 306 patients with stage I NSCLC from four institutions in Japan, with a median age of 75 years. The radiotherapy dose was 52.8–64.0 Gy/4 fr in 181 patients, a single fraction of 46.0–50.0 Gy in 97, and other doses in 28 patients. The results showed that the 3-year overall survival rate, progression-free survival rate, and local control rate were 83.6%, 69.4%, and 88.6%, respectively [51]. For stage II and III NSCLC, Karube et al. investigated 64 patients at three institutions. The 2-year overall survival rate was 62.2%, the 2-year progression-free survival rate was 42.3%, and the local control rate was 81.8% [4]. For a long time, CIRT has rarely been recommended for patients with stage III NSCLC because there is insufficient evidence of its effectiveness. In recent years, there has been increasing evidence of its efficacy in Japan for stage III NSCLC [52]. Saitoh et al. treated six patients with CIRT at a dose of 4 Gy each time and a total dose of 64 Gy. The results showed that no patients had grade ≥ 3 toxic reactions during the treatment, and all patients achieved tumor control during the survival period. These results demonstrate that hypofractionated CIRT is feasible in the treatment of stage III NSCLC with small adverse reactions, which also supports further research [53]. Anzai et al. studied 65 patients with stage III NSCLC who received a median dose of 72 GyE. The 2-year local control rate was 73.9%, the 2-year progression-free survival rate was 38.6%, and the overall survival rate was 54.9%. Although 1 (2%) and 5 (8%) patients developed grade 4 and grade 3 toxicities, respectively, the authors considered these probabilities to be acceptable [54]. The results also suggest the potential of heavy ion radiotherapy in the treatment of advanced NSCLC. Platinum-based concurrent chemoradiotherapy has been the standard for NSCLC patients who cannot be treated surgically. However, the results were far from ideal. In recent years, the “PACIFIC regimen”, consisting of 12 months durvalumab after chemoradiotherapy, has attracted global attention. Durvalumab is a selective and high affinity human immunoglobulin monoclonal antibody that blocks the binding of PD-L1 to PD-1 and CD80, fights tumor immune escape, and triggers immune responses in the body. Faivre-Finn et al. conducted the placebo-controlled Pacific trial in 709 patients with stage III NSCLC. It was found that the estimated 4-year overall survival for durvalumab versus placebo was 49.6% versus 36.3%, and the estimated 4-year progression-free survival was 35.3% versus 19.5%, respectively [55]. Considering the larger immunostimulatory capacity of carbon ions over photons as mentioned above, the combination of PACIFIC regimen with CIRT may be promising for advanced NSCLC.

Within a certain range, the higher the radiation dose, the more effective the treatment, and the more damage to normal tissue. The choice of dose often depends on the stage of the tumor and the individual situation of the patient [56]. Yamamoto et al. conducted a dose escalation trial of single-fraction CIRT for stage I NSCLC. They followed 218 patients who received CIRT with doses ranging from 28 to 50 Gy. Among them, the median age of patients was 75 years. A total of 123 patients had stage T1 tumors, and 95 patients had stage T2 tumors. The 5-year overall survival rate was 49.4% and the local control rate was 72.7%. There was a statistically significant difference in local control rates between patients receiving 36 Gy or more and those receiving less than 36 Gy. The 5-year local control rate was 95.0%, and the overall survival rate was 69.2% in 20 patients exposed to 48–50 Gy carbon ion beams. There was only one with grade 3 toxicity in these patients, and the proportion of grade 2 toxicity was less than 2% [57]. Regardless of photon SBRT, proton radiotherapy, or CIRT, multiple fraction radiotherapy was used in the beginning, but now single fraction high-dose radiotherapy is also widely used. Ono et al. also studied the treatment of early NSCLC with 50 Gy single fraction CIRT. Fifty-seven patients with a median age of 75 years (42–94 years) were treated and followed for a median period of 61 months (6–97 months). The 3-year and 5-year overall survival rates were 91.2% and 81.7%, respectively. The 3-year and 5-year local control rates were 96.4% and 91.8%, respectively. There were no cases of ≥grade 3 pneumonitis [58]. In conclusion, high-dose single fraction CIRT has good efficacy, and the adverse reactions are within the acceptable range. Multiple fraction radiotherapy has good efficacy and few serious adverse reactions, but it requires a long treatment cycle and high cost. Single fraction therapy requires a short treatment time and relatively low cost without loss of efficacy; however, it is necessary to bear the adverse reactions that may be caused by large doses of radiation [57]. For stage III and above NSCLC, conventionally fractionated radiotherapy (1.8–2.0 Gy per day, total dose 60–70 Gy) combined with chemotherapy is the standard treatment over a long time, but the outcomes are poor, and local recurrence is common. The overall survival rate is less than 20% [59]. Bradley et al. studied the treatment results under different doses of 60 Gy and 74 Gy, and the results showed no significant difference, and the high dose of radiation in normal tissue had potential harm [60]. This result could also be interpreted as indicating that the damage caused by high doses of radiation offsets its effects. There are still many questions to be answered about the relationship between dose and efficacy. For example, the most serious problem is local recurrence. Low-dose radiation is a key factor in the recurrence of NSCLC tumors [61], and approximately 10% of patients experience local recurrence within 5 years [58]. For some lesions, the therapeutic dose is not good enough to control the tumor, so appropriately increasing the central dose or combining it with chemotherapy drugs is a possible solution.

Patient individual factors, such as age and underlying diseases, will also affect the results of CIRT. Hayashi et al. studied 32 patients over 80 years of age. The median age of these patients was 82 years (age range 80–88 years), with 17 stage II patients and 15 stage III patients. All patients completed CIRT, including 1 case of grade 3 toxicity, seven cases of grade 2 toxicity, and no grade 4 toxicity. The two-year local control rate, progression-free survival rate, and overall survival rate were 83.5%, 46.7%, and 68.0%, respectively. This study demonstrates that CIRT is safe and effective in NSCLC patients over 80 years of age [62]. Many elderly patients with NSCLC also suffer from other diseases such as chronic obstructive pulmonary disease (COPD), interstitial lung disease (ILD) [63], and cardiovascular disease (CVD). These patients are generally medically inoperable [64]. Sugane et al. used CIRT to treat 28 inoperable patients over 80 years of age with stage I NSCLC. The 5-year local control rate and survival rate were 95.8% and 30.7%, respectively. This result suggests that CIRT is effective in elderly inoperable patients with NSCLC [65]. Shioyama et al. conducted a retrospective study on CIRT for stage I NSCLC and analyzed the difference in treatment results between patients who could undergo surgery and those who could not. There were 306 patients in total, 139 of whom were considered inoperable. The results showed that the 3-year overall survival rate and progression-free survival rate were 90% and 76%, respectively, in the operable patients, and 76% and 62%, respectively, in the inoperable patients. The treatment outcome of operable patients was significantly better than that of inoperable patients [51]. ILD is a risk factor. Radiation may worsen ILD or cause severe radiation pneumonia [66]. Okano et al. analyzed the results of 124 patients treated with CIRT for stage I NSCLC and divided them into an ILD group (26 patients) and a non-ILD group (98 patients). The 3-year overall survival rate was 83.2% in the non-ILD group and 59.7% in the ILD group. Radiation pneumonitis severer than grade 2 was observed in three patients (3.0%) in the non-ILD group and two patients (7.6%) in the ILD group. The results showed that CIRT was feasible for the treatment of stage I NSCLC in both ILD and non-ILD groups, and ILD was a high-risk factor for stage I NSCLC [67]. Nakajima et al. treated 29 patients with NSCLC with ILD, ranging in age from 62 to 90 years. The 3-year local control rate and survival rate were 63.3% and 46.3%, respectively, and seven patients developed exacerbation of symptoms. These results suggest that CIRT may be a low-risk therapy for NSCLC patients with ILD who are not suitable for conventional therapy [63].

In addition, it is difficult to treat patients with recurrent NSCLC [68]. CIRT also shows some potential in this area. Karube et al. used CIRT to treat 29 patients with recurrent NSCLC, whose median age was 74 years. The 2-year overall survival rate, local control rate, and progression-free survival rate were 69%, 66.9%, and 51.7%, respectively. No case of ≥grade 3 toxicity was recorded [69]. These results suggest that CIRT can be used as a treatment for recurrent NSCLC. The CIRT treatment cases of different stages of NSCLC were summarized in Table 1.

## 6. Discussion

Although CIRT achieved excellent efficacy in the treatment of NSCLC, it still has some shortcomings. The first is the inevitable radiation damage. A common and serious injury is radiation pneumonia [72]. Although radiation pneumonia is relatively rare with heavy ion radiotherapy compared with X-rays, it can still be dangerous. Chromosomal aberrations in lymphocytes induced by radiation are also common adverse reactions [73,74,75]. Lee et al. studied 22 lung cancer patients who received different doses of carbon ion irradiation and found that the proportion of lymphocytes with chromosomal aberrations in the peripheral blood increased by approximately 4%. Despite obvious individual differences, it is still a risk factor that cannot be ignored [76]. In addition, adverse reactions such as radiation esophagitis, radiation tracheitis, bone marrow suppression, and radiation-induced fracture are also factors that need attention [8]. Second, the effect of respiratory movement on the treatment of lung cancer cannot be ignored [77,78]. Due to respiratory movement, the location and thickness of the lesion will change, and this effect is unavoidable [79]. Third, as the clinical treatment of NSCLC with heavy ions has not been long enough, there is no adequate sample size to support some speculations [80]. In addition, in some studies, the individual differences of patients (age, underlying diseases, etc.) may have a great impact on experimental results due to the small sample size, resulting in limited availability of research conclusions. Fourth, the cost is high. The installation cost of carbon ion devices is approximately 140 million dollars, while the installation cost of proton devices is approximately 70 million dollars [81]. Besides, the average cost of CIRT is approximately 50,000 dollars, while the treatment cost of SBRT is approximately 15,000 dollars [82]. The high cost of treatment has become an obstacle for many patients.

In our opinion, there are some possible solutions to these problems. (1) Combine CIRT with imaging techniques such as PET/CT [12,83,84,85]. For example, using four-dimensional cone beam computed tomography (4D-CBCT) reconstructed by simultaneous motion estimation and image reconstruction (SMEIR) can capture the projections of all phases of lungs on the treatment day. Therefore, better treatment planning and dose calculation can be performed for CIRT, and the effect of periodic lung movement on the curative effect will be reduced [83,86,87]. In addition, the Compton camera is capable of detecting 511 keV annihilation gamma rays produced by carbon ion beams interacting with target tissues and is small enough to be easily installed in a treatment room. Therefore, the determination of the peak intensity and beam range of the carbon ion beams by Compton camera may reduce the dose distribution degradation during treatment, which would contribute to the more accurate and effective application of CIRT in cancer treatment [88]. (2) Adjuvant chemotherapy drugs related to heavy ion radiotherapy should be studied to improve the efficacy [89,90,91,92,93,94]. Ma et al. studied 25 patients with small cell lung cancer who received proton and carbon ion combined radiotherapy. Among them, the median dose of protons and carbon ions was 67.1 Gy, and chemotherapy drugs included etoposide and platinum, for a total of four to six cycles. Radiotherapy and chemotherapy were carried out simultaneously. The 2-year overall survival rate was 81.7%. The progression-free survival rate was 66.7%, and five patients (20.0%) had grade 3 adverse reactions. These results have implications for combination therapy [95]. (3) A combination of immunotherapy and CIRT may improve the efficacy. Although there has not been enough clinical data to prove the efficacy of the combination of CIRT with immunotherapy in the treatment of NSCLC, it was found that, combined treatment with carbon ions and immune checkpoint inhibitors reduced the lung metastases efficiently in a syngeneic murine osteosarcoma model while radiation or checkpoint inhibitors alone were not sufficient to reduce the growth of the abscopal tumors, suggesting immense potential of the combination of immunotherapy and CIRT [96]. Besides, durvalumab, as an immune checkpoint inhibitor, has been proved effective in the control of advanced NSCLC after concurrent chemoradiotherapy [55], whose efficacy deserves further verification when combined with CIRT. (4) Actively carry out cooperation among multiple organizations, and carry out large-scale multicenter prospective research on heavy ion therapy, which is of great significance for clinical application. (5) Seek technological breakthroughs and industrial innovation. In the treatment cost of CIRT, the technology cost is approximately $28,000, so reducing the technology cost is of great importance. In addition, Okazaki et al. compared CIRT with SBRT in terms of cost-effectiveness for treating stage I NSCLC and found CIRT is a more cost-effective treatment approach. The incremental cost-effectiveness ratios (ICER) were 64,891 dollars/life year and 32,123 dollars/life year for all patients and propensity score-matched patients, respectively. Hospitalization and examination costs were significantly higher in the CIRT group and even showed greater impact on ICER than technical costs, implying that reasonable planning of examination frequency and hospitalization time are also the directions of reducing treatment costs [82].

According to the current tumor prevalence, NSCLC is still a major threat to human health. Since conventional radiotherapy combined with chemotherapy has little effect on NSCLC, we summarize the research progress of heavy ion radiotherapy for this kind of lesion, aiming to provide an effective way for patients to achieve a good therapeutic efficacy. In recent years, heavy ion treatment technology has developed rapidly. Japan, Germany, Italy, China, and Austria have their own heavy ion radiotherapy centers, and France, South Korea, the United States, and other countries are also actively preparing to construct heavy-ion therapy centers. Cooperation between countries and institutions is becoming increasingly close, such as the establishment of tumor proton therapy centers for international cooperation between China and the United States. Currently, there are two heavy ion hospitals in operation in China (mainland), namely, Shanghai Proton and Heavy Ion Center and Heavy Ion Center of Wuwei Cancer Hospital, while a number of regions are preparing to build heavy ion treatment centers.

Although heavy ion radiotherapy has a considerable curative effect, there are still many problems to be solved if we want to popularize it to more patients and expand its application fields, such as the optimization of the dose division method, the application and optimization of respiratory gating technology, and the combination with other therapies. The solution of these problems depends on generous efforts from both radiobiologists and clinical radiologists. It is believed that in the future, heavy ion radiotherapy will have broader application prospects for NSCLC and other tumors.

## Figures and Tables

**Table 1:** Summary of treatment cases of different stages of NSCLC by CIRT.

Tumor Staging	Sample Size	Dose/Fractions	Overall Survival	Local Control	Toxicity	Reference
I	50	72 Gy/9 fr	5-year, 50%	5-year, 94.7%	Grade ≥ 3: 0 (0%)	[70]
I	79	52.8 Gy/4 fr for stage IA 60.0 Gy/4 fr for stage IB	5-year, 45%	5-year, 90%	Grade ≥ 3: 0 (0%)	[48]
I	218	28–50 Gy/1 fr	3-year, 68.3% 5-year, 49.4%	3-year, 77.9% 5-year, 72.7%	Grade ≥ 3: 1 (0.46%), Grade 3 chest wall pain: 1 (0.46%)	[57]
I	57	50 Gy/1 fr	3-year, 91.2% 5-year, 81.7%	3-year, 96.4% 5-year, 91.8%	Grade ≥3: 0 (0%)	[58]
I/II	47	59.4–95.4 Gy/18 fr	5-year, 42%	5-year, 64%	Grade ≥ 3: 3 (3.7%), Grade 3 radiation pneumonitis: 3 (3.7%)	[71]
I/II	34	68.4–79.2 Gy/9 fr		5-year, 84%		
II/III	32	68.0–76.0 Gy/12–16 fr	2-year, 68.0% 3-year, 54.3%	2-year, 83.5% 3-year, 77.1%	Grade ≥ 3: 1 (3.1%), Grade 3 radiation pneumonitis: 1 (3.1%)	[62]
II/III	64	52.8–72.0 Gy/4–16 fr	2-year, 62.2%	2-year, 81.8%	Grade ≥ 3: 0 (0%)	[4]
III	65	64.0–76.0 Gy/16 fr	2-year, 54.9% 3-year, 42.0%	2-year, 73.9% 3-year, 70.2%	Grade ≥ 3: 6 (9.2%), Grade 3 radiation pneumonitis: 4 (6.2%), Grade 3 bronchial fistula: 1 (1.5%), Grade 4 mediastinal haemorrhage: 1 (1.5%)	[54]

## Data Availability

Not applicable.
